# Comparing the Performance of Bread and Breakfast Cereals, Dairy, and Meat in Nutritionally Balanced and Sustainable Diets

**DOI:** 10.3389/fnut.2018.00051

**Published:** 2018-06-07

**Authors:** Gerard F. H. Kramer, Elsa V. Martinez, Namy D. Espinoza-Orias, Karen A. Cooper, Marcelo Tyszler, Hans Blonk

**Affiliations:** ^1^Blonk Consultants, Gouda, Netherlands; ^2^Nestlé Research Center, Lausanne, Switzerland

**Keywords:** sustainable diets, quadratic optimization, nutrient balance, environmental impact, bread, breakfast cereals, dairy, meat

## Abstract

**Objective:** To quantify the performance of food products in a sustainable diet based on the balance of their contribution to nutrient intake and environmental impact, within the context of the Dutch diet.

**Design:** While fixing the quantity of a specific food group at different levels, optimized diets that met nutrient requirements and stayed as close as possible to the current Dutch diet were calculated, in order to understand its potential environmental impact and its nutritional quality. Bread & breakfast cereals, dairy, and meat were compared between 0 and 250% of current intake. Their performance is expressed in the relationship between the quantity of these food products and (1) the environmental impact of diets and (2) the nutrient balance of the diets.

**Setting:** The Netherlands.

**Subjects:** Women aged 31–50.

**Results:** The amount of bread & breakfast cereals in the optimized diets were inversely correlated with their environmental impact. The nutrient balance of the optimized diets was maintained despite varying cereal content, with the expected improvement over the current diet. Increasing amounts of dairy in the optimized diet were associated with an increase in environmental impact and meat with a steep increase. The nutrient balance of optimized diets with varying dairy and meat contents was also maintained at high levels, even at 0% content.

**Conclusions:** Bread and breakfast cereals are sources of nutrients with a better environmental performance compared to dairy or meat within the context of the Dutch diet. It is possible to optimize diets for environmental impact whilst maintaining a high nutrient balance.

## Introduction

It is evident that the food system has an important role in global greenhouse gas emissions (1, 2) and the depletion of natural resources (3). This has led to an increased scientific interest in the sustainability of diets. It is widely recognized that many diets need to change to reduce the environmental impact (4, 5). How this can be done, while respecting nutritional quality and local dietary habits, is the focus of an increasing number of studies (6–8). These studies showed that the performance of specific food products in sustainable diets is determined by the balance between their nutritional quality and environmental impact.

In particular, for food manufacturing companies, it is important to know how their current food products contribute to a sustainable diet and how the performance of new food products can be optimized. Several metrics have been suggested to capture this integral performance (9, 10) ideally combining a metric for nutritional quality or nutrient density with environmental metrics from Life Cycle Assessment (LCA).

Methodology in this area is evolving rapidly. We contribute to this developing research field by presenting a method that evaluates the balance between nutritional quality and environmental impact within the context of a total diet. Relative to the current diet, we show the effects of increasing and decreasing the intake of a specific food product or food group on the environmental impact of the total diet. This is done by optimizing the current diet iso-calorically so that nutritional requirements are met while holding the amounts of the food (group) of interest fixed at different levels. Consequently, other food products in the diet will either be replaced or have their amounts changed. If an optimized diet with increased amounts from a food (group) of interest has a lower environmental impact and a better nutrient profile, the food (group) can be regarded as a source of nutrients with a better environmental performance than the food products replaced or changed. We optimize the diets by minimizing the changes to the current diet (11, 12) until all nutrient requirements are met. We use a current Dutch diet as starting point and examine the performance of three food groups: bread and breakfast cereals, dairy, and meat. These three groups are key food groups in the average Dutch diet and have recently been subject to question about their role in a sustainable and/or healthy diet in both scientific and gray literature. Dairy and meat are typically questioned about their high environmental impact, even though they are sources of high biological value proteins, while bread & breakfast cereals have positive recommendation from public health authorities (13), in contrast with negative recommendation from popular media (14, 15).

The above exercise revealed that bread and breakfast cereals has the best environmental performance of the three groups, whilst maintaining a good nutrient balance. The exercise was not exhaustive and did not compare the performance other groups, which could be an extension of the present study.

## Methods

Starting from a current diet, the amounts of a food group of interest were fixed in varying levels in steps of 25% between 0 and 250% of the quantity in the current diet. In each of the 11 steps, after fixing the amount of the food group of interest, the diet was iso-calorically optimized. Notice that within the food group of interest changes are also allowed, as long as the total mass in grams stays at the set level. In the case of dairy, the current diet ratio of liquid dairy products to cheese was kept constant at 8.76 g:1 g in order to avoid artificial environmental improvements by substituting cheese for milk. The exercise resulted in 11 iso-caloric optimized diets satisfying nutritional requirements and with associated nutritional, environmental, and optimization metrics.

We detail below the elements of the optimization and metrics computed.

### Current diet

An average weekly diet (current diet) for Dutch women aged 31–50 was derived from the Dutch National Food Consumption Survey (DNFCS, 2007–2010) (16, 17). The procedure is described elsewhere (18). The analysis included a database of a total of 208 food products containing both nutritional composition (19) and environmental impacts. These are representative for food products consumed nowadays in the Netherlands (20–22), excluding brands. The list contained a limited number of fortified products, such as soy drink, meat replacer, and (iodine fortified) bread. Cereals included were mainstream food products and not fortified (19).

### Environmental impacts

Life Cycle Assessment (LCA) methodology (23) was applied to calculate the environmental impacts (20, 24) [Greenhouse Gas Emissions (GHGe), Fossil Energy Use (FEU), and Land Occupation (LO)] associated with each of the 208 food products and the optimized diets. The source database is of high quality and been reviewed externally by Centre for Design and Society, RMIT University, Melbourne, Australia, and by the Netherlands National Institute for Public Health and the Environment (23). The scope of the LCAs in this study included agricultural production (in the Netherlands and abroad), transport, processing, distribution, retail (lighting, cooling), consumer phase (e.g., cooling and cooking), and waste treatment. The Carbon Footprint for a selection of products is shown in Table 1.

**Table 1:** Carbon Footprint of a selection of products available in the diet.

**Product**	**GHGe**
	**(kg CO_2_-eq/kg)**
Beef	46.7
Cheese, Gouda	9.2
Pork	7.7
Chicken	5.1
Salmon	3.9
Egg	3.3
Herring	2.0
Tomato	1.7
Cashew nuts	1.6
Milk, semi-skimmed	1.2
Crispbread	1.0
Bread, white	1.0
Bread, rye	0.9
Bread, wholemeal	0.9
Carrots	0.7
Potatoes	0.7
Cereal, wholegrain	0.7
Apple	0.5

*These results were calculated by the authors for the purposes of this article*.

### Nutrient balance concept (NBC)

The NBC (10, 25) is a nutrient profiling concept that evaluates the nutritional values of multiple food products in meals and total diets. The NBC advances on nutrient density by adding the metric of nutrient balance (NB) to qualifying (QI) and disqualifying (DI) indices (25). The QI is defined as the ratio of 28 essential nutrients contained in 2,000 kcal of a given food product relative to the country reference intakes for those nutrients. The DI is defined as the ratio of 7 public health sensitive nutrients contained in 2,000 kcal of a given food product, relative to the Maximal Reference Values (MRV) for those nutrients. If the QI value is >1, the food product is considered nutrient dense; if the QI value is smaller than 1, the food product is considered energy dense. If the DI value is >1, the food product is deemed compromised because it contains disqualifying nutrients in values higher than the MRV relative to the energy content of the food product. Finally, the NB score is calculated as the average proportion of daily values for qualifying nutrients (QI) present in 2,000 kcal of a given food, truncated at 1 for each qualifying nutrient. A NB score of 100% is achieved if every qualifying nutrient satisfies 100% or more of its daily requirement. Table 2 summarizes the calculations of QI, DI, and NB.

**Table 2:** Calculation of QI, DI, and NB (25).

**Equations**	**Description**	**Nutrients considered**
$\text{QI}=\frac{{\text{E}}_{\text{d}}}{{\text{E}}_{\text{p}}}\cdot \frac{\sum_{\text{j = 1}}^{{\text{N}}_{\text{q}}}\frac{{\text{a}}_{\text{q},\text{j}}}{{\text{r}}_{\text{q},\text{j}}}}{{\text{N}}_{\text{q}}}$	E_d_ = daily energy need (kcal) E_p_ = energy in the qtty. of food analyzed (kcal) a_q,j_ = qtty. of qualifying nutrient (g) a_d,j_ = qtty. of disqualifying nutrient (g) r_q,j_ = DRI of qualifying nutrient (g/day) r_d,j_ = MRV of disqualifying nutrient (g/day) N_q_ = Number of qualifying nutrients evaluated N_d_ = Number of disqualifying nutrients evaluated QI = Qualifying index DI = Disqualifying index NB = Qualifying nutrient balance score	**For QI**: folate; niacin; panthotenic acid; riboflavin; thiamin; vitamins A, B6, B12, C, D, E, K; Ca, Cu, F, Fe, Mg, Mn, K, P, Se, Zn; α-linolenic acid; linolenic acid; choline; dietary fiber; protein; water. **For DI**: total fat; saturated fat; cholesterol; trans fatty acids; total sugars; Na; alcohol.
$\text{DI}=\frac{{\text{E}}_{\text{d}}}{{\text{E}}_{\text{p}}}\cdot \frac{\sum_{\text{j = 1}}^{{\text{N}}_{\text{d}}}\frac{{\text{a}}_{\text{d},\text{j}}}{{\text{r}}_{\text{d},\text{j}}}}{{\text{N}}_{\text{d}}}$		
$\text{NB }=100\cdot \frac{\sum_{\text{i = 1}}^{{\text{N}}_{\text{q}}}{\text{QI}}_{\text{i}}}{{\text{N}}_{\text{q}}}$		

To compute NBC metrics comparable with previous NBC studies, the 208 food products in these diets were matched with an equivalent food from the USDA Food Composition Database (Release 28).

### Diet optimization

The Optimeal® software (Blonk Consultants, Gouda, The Netherlands) was used to compute the diets most similar to the current diet and simultaneously satisfying the set of specified nutritional requirements. This is known as quadratic programming. As described previously (18, 26), the Optimeal® software contains all required data on food composition (19), nutrient requirements (27, 28), and environmental impacts of food products consumed in the Netherlands.

In the model, the nutritional requirements are: (a) the Dutch (27) Recommended Dietary Allowance (RDA) or Adequate Intake (AI) (when an RDA cannot be determined), defined as lower limits; and (b) the Tolerable Upper Intake Level (UL), defined as upper limit (see Supplementary Table S1). The sugar limit used in the NBC metrics was taken from the Institute of Medicine (29) as no formal recommendation on total sugars is provided in the Netherlands, only on total carbohydrates. To avoid confounding effects, all diets were iso-caloric with 1,995 kcal per day.

The similarity of two diets can be measured by the Euclidian distance (ED) between them, i.e., by the square root of the sum of the square differences (in grams) of the amounts of each food product in the two diets. The Euclidian distance is the generalization of the shortest path between two points. It is assumed that a similar diet is easier to adopt by consumers than one that is more deviating from their current diet. At a population level, shifts in consumption of food products are relatively gradual.

## Results

**Figure 1:**
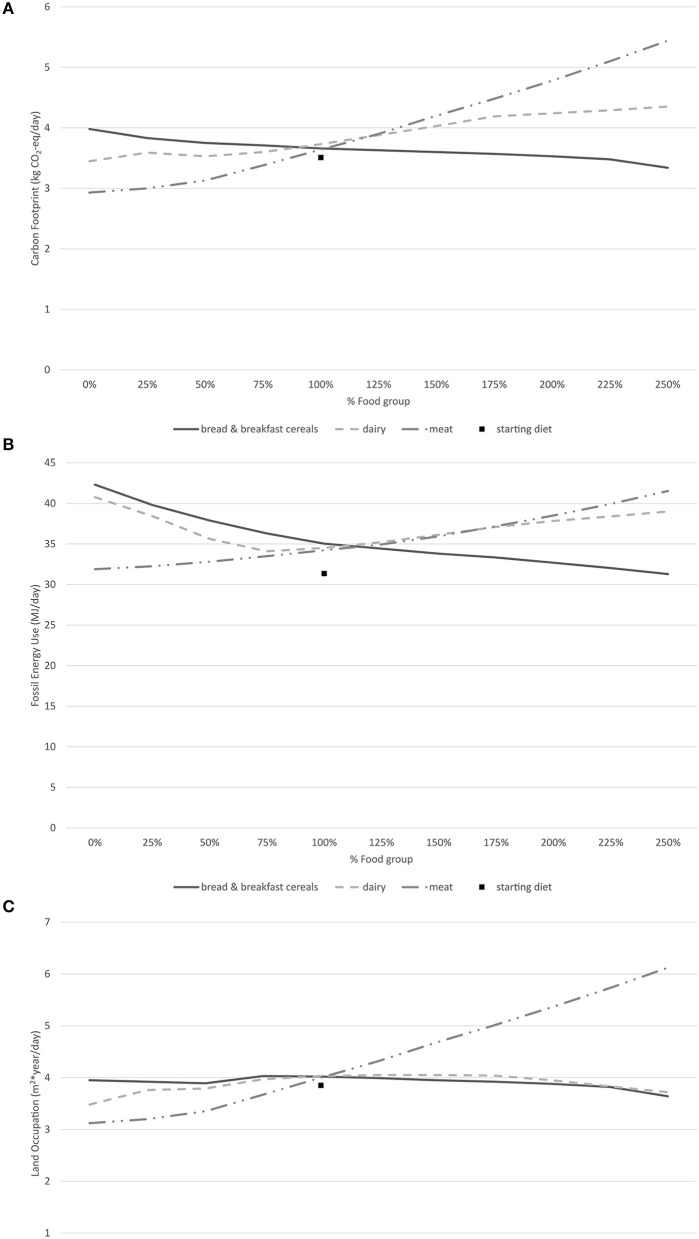
Environmental Indicators with varying amounts of dairy, meat and bread and breakfast cereals, relative to the current intake of each (100%). **(A)** Carbon footprint. **(B)** Fossil energy use. **(C)** Land occupation. These results were calculated by the authors for the purposes of this article.

**Figure 2:**
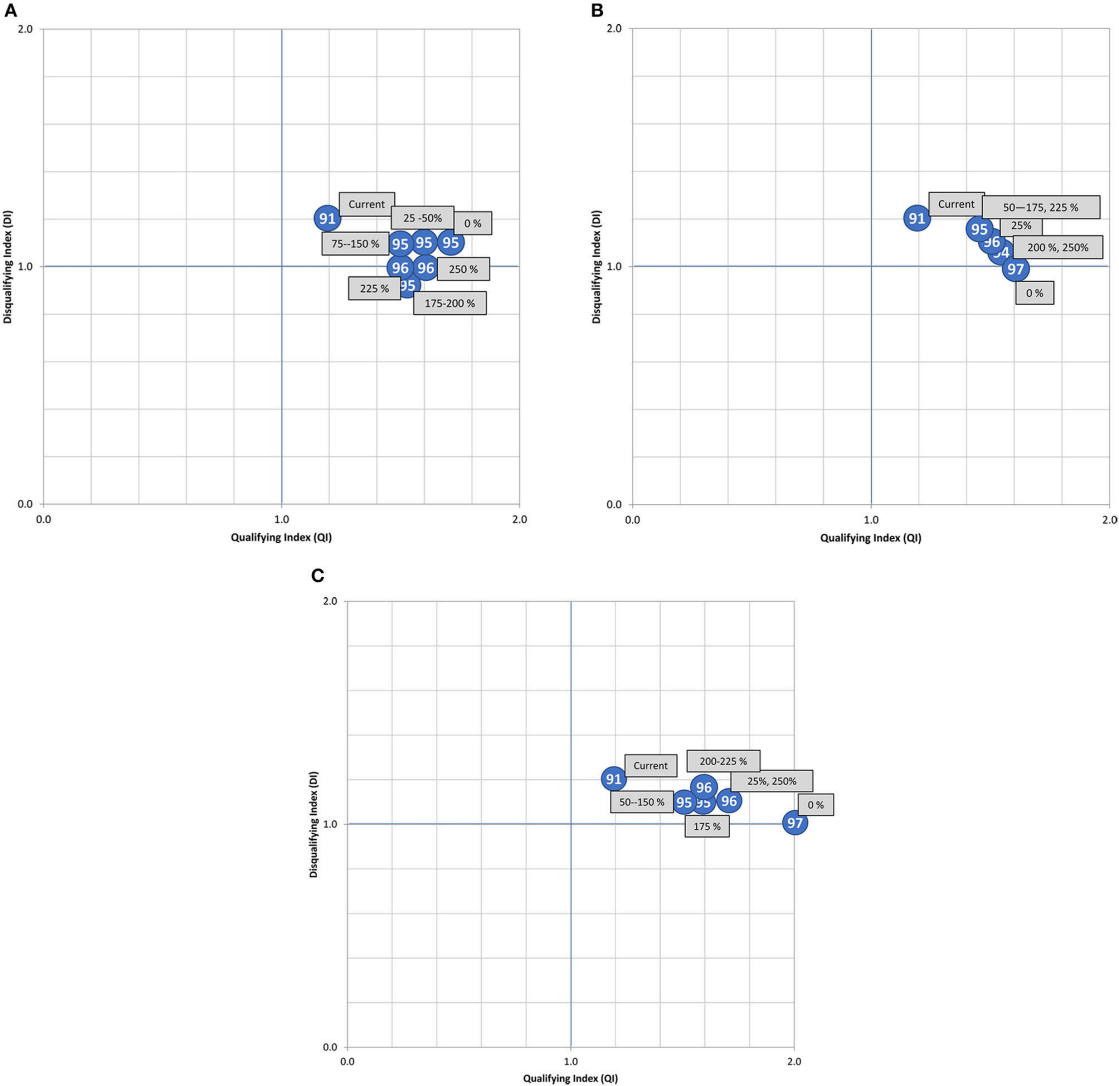
QI, DI, and NB (shown in the blue circles) for the optimized diets. **(A)** Bread and breakfast cereals. **(B)** Meat. **(C)** Dairy. These results were calculated by the authors for the purposes of this article.

The results of the assessment of the three food groups are shown graphically in Figure 1. The slopes in the graphs with environmental impacts are indicative of the environmental performance of a food group as a source of nutrients. Figure 2 shows the NB, QI, and DI of the optimized diets, being indicative of the nutrient balance.

Overall, increased amounts of meat products were associated with higher environmental impacts. Relative to the current diet, GHGe, FEU, and LO increased 49, 21, and 53%, respectively at the 250% level. In contrast, when the content of meat in an optimized diet was reduced, the environmental impacts decreased rapidly and attained the lowest values observed in this study. The NB score ranged from 94.5 to 97.1 with the meat content variation. Notably, the best NB score was achieved with the 0% meat level. At this level, the optimized diets also had the lowest DI and highest QI, suggesting a potentially positive balance can be achieved through dietary optimization after removal of meat products from the diets.

The optimized diet with 250% level of current dietary dairy resulted in an increase of 16% in GHGe and 13% in FEU relative to the current diet, but in a 9% reduction of LO. The latter coincides with a shift in the type of land use from cropland to pasture, which, in general, is associated with a lower environmental impact. The NB score also varied from 94.8 to 96.6. The optimized diet with 0% dairy also had the highest QI overall, and the lowest DI from the dairy-optimized diets.

The results show that bread and breakfast cereals had the lowest impact when its amounts were increased. The optimized diet with 250% level of the current level of bread and breakfast cereals reduced 14% in GHGe, 12% in FEU, and 15% in LO, compared to the current diet. In contrast, decreasing bread and breakfast cereals resulted in the highest environmental impact compared to the other two food groups studied. Overall, the amounts of bread and breakfast cereals were inversely correlated with the levels of GHGe and FEU. Both increased and decreased amounts of bread and breakfast cereals were associated with lower levels of LO. The NB score varied from 94.9 to 96.0, QI varied from 1.5 to 1.7 and DI from 1 to 1.1, indicating a small positive relationship between the amounts of bread and breakfast cereals and the nutrient balance of the diet.

Since bread and breakfast cereal was the food group with better performance, we investigated it in more detail. Table 3 shows the changes in the most important food groups in the 11 optimized diets with varying amounts of bread and breakfast cereals. Among the nutrients in bread and breakfast cereals that have to be replaced at 0% (see Supplementary Table S1) are total energy, iodine, fiber, and vitamin B1. This explains increased amounts of fish (iodine), legumes (energy, fiber, B1), pasta/rice (energy, B1), and vegetables (fiber, B1).

**Table 3:** Shifts in food groups for different percentages of bread and breakfast cereals (women 31–50 years) relative to current intake (=100%).

**Food Group [g/day]**	**Starting diet**	**0%**	**25%**	**50%**	**75%**	**100%**	**125%**	**150%**	**175%**	**200%**	**225%**	**250%**
**Bread and Breakfast Cereals**	**141**^a^	**0**	**35**	**70**	**106**	**141**	**176**	**211**	**245**	**282**	**317**	**352**^b^
Cheese	37	17	18	20	21	22	21	21	22	22	22	21
Eggs	11	16	14	13	13	12	12	13	13	14	14	14
Fish	17	92	73	52	36	28	28	27	27	27	27	29
Fruits	120	175	167	160	152	145	139	133	126	118	109	102
Legumes	4	68	61	54	46	39	33	28	22	16	9	7
Meat	88	69	74	79	85	86	86	85	84	82	76	59
Dairy	328	335	328	321	317	312	311	310	309	307	302	296
Pasta/Rice	52	60	58	56	55	53	52	51	49	46	43	39
Vegetables	134	381	352	329	299	274	254	237	222	206	191	196

a *141 g consists of 0.6 tablespoons of cereals, 0.4 slices of crispbread, and 3.5 slices of bread*.

b *352 g consists of 8.4 tablespoons of cereals, 4.7 slices of crispbread, and 6.5 slices of bread*.

*These results were calculated by the authors for the purposes of this article*.

It is also relevant to understand the changes within the food group. Table 4 presents the ratio of wholegrain to other varieties of bread and breakfast cereals. In the current diet the ratio was 0.69. Absolute amounts of both wholegrain and other varieties increase as the total amount of bread and breakfast cereals increase. The ratio of wholegrain to other varieties, however, decreases.

**Table 4:** Ratio of wholegrain to refined with different amounts of Bread and breakfast cereals.

**Diet**	**Wholegrain (g)**	**Refined (g)**	**Ratio Wholegrain:refined**
Starting diet	57	83	0.69
0%	0	0	0.00
25%	24	12	2.04
50%	39	31	1.26
100%	80	61	1.31
150%	112	99	1.12
200%	138	144	0.96
250%	168	184	0.92

*These results were calculated by the authors for the purposes of this article*.

## Discussion

The relative order in performance of meat, dairy and bread and breakfast cereals obtained can be explained by the balance between nutritional performance and environmental performance. In particular, wholegrain cereal products combine a high nutrient balance (25) score with a low environmental impact, whereas meat combines a medium nutrient balance score with a high environmental impact (30). It is important to realize, however, that bread is an iodine fortified product. Dairy has an intermediate position in both metrics (nutritional and environmental performance), which is in agreement with previous studies (6, 31). This observation can also be explained by the fact that cheese and liquid dairy were introduced in a fixed ratio.

The NB scores vary minimally with changes in cereals content, due to the fact that all the optimized diets were high scoring, i.e., above 90%, and so in order to facilitate an increase, the changes in cereals and resultant overall optimized diets would need to address the small undersupply of just a few nutrients, which in this case were fiber, choline, vitamin E, and vitamin D. It is possible that if the wholegrain content of the optimized diets had been increased at the same rate as total cereals, some of these shortfall nutrients might have been met (fiber, vitamin E, iron). Interestingly, iron was undersupplied in the optimized diets with <150% of current intake of bread and breakfast cereals. However, in all the optimized diets, fat and sugar were oversupplied. The fact that the NB score of the optimized diets was always very high (circa 94–95%), is not surprising, as they are all optimized to meet Dutch nutrient recommendations.

**Figure 3:**
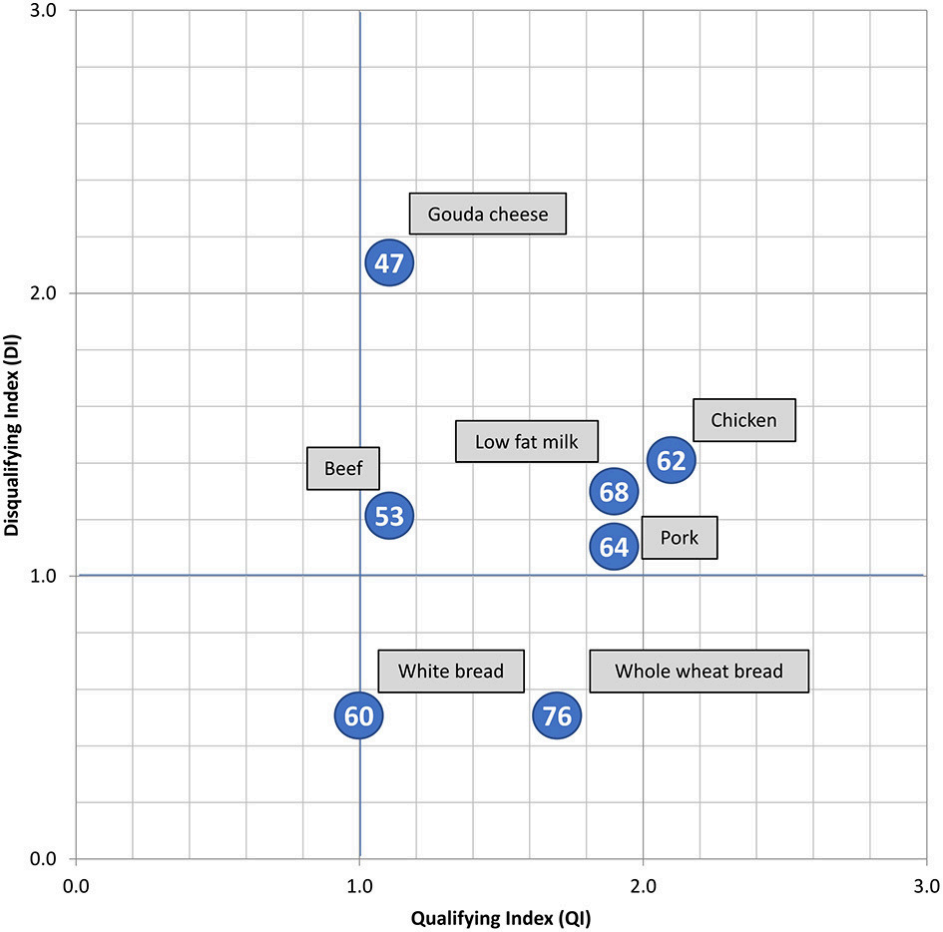
Exemplar products to demonstrate relative positions and scores from the Nutrient Balance Concept analyses. The NB scores per product are shown in the blue circles. These results were calculated by the authors for the purposes of this article.

NB scores were also calculated for exemplar foods from each of the three food groups of interest (Figure 3). Results show that whole wheat bread and low-fat milk had a higher NB score, and Gouda cheese and beef a lower NB score. Differences in contents of dietary fiber, saturated fat and salt explain most of the differences, for instance between white bread and whole wheat bread. The different types of meat, beef in particular, performed worse than both types of bread. As for dairy, low-fat milk performed much better than Gouda cheese.

Even though all optimized diets are plausible in the sense that they satisfy all nutritional requirements and are as close as possible to the current diet, attainability is a question. In earlier studies (18, 26) with the Optimeal® tool, Linear Programming was applied, i.e., the distance between two diets was measured by a linear transformation, the sum of the absolute differences of the normalized amounts. The disadvantage of that technique is that it tends to prefer large shifts in a limited number of food products in the diet, for instance by adding large quantities of legumes (18). This seems unrealistic at population level, as shifts in national diets tend to be gradual. By minimizing the Euclidian Distance, large shifts in a single food are more heavily penalized, resulting in more realistic solutions.

Moreover, the proposed method provides insight in the attainability of changes, by investigating distance between diets. This analysis shows that meat seems to be replaced more easily than bread and breakfast cereals and dairy. As mentioned already, bread is an iodine fortified product. Studying the effects of (iodine) fortification was out of scope and is a limitation of this study. Replacement of dairy is more difficult to attain since these products are nutrient dense and it would require more additional food products to be included in the optimized diet to provide the missing nutrients such as calcium and vitamins B2 and B12. In most cases these alternative products are not as nutrient dense as dairy. This reinforces the advice that a varied diet is the best way to achieve adequate nutrient intake.

This study is limited by the environmental data. Only GHGe, EU, and LO were analyzed, ignoring other environmental impacts. For example, the reduction of LO at lower levels of bread and breakfast cereals can be explained by the replacement of bread and breakfast cereals by fish, which is one of the few other food sources of iodine. Wild-caught fish has no LO, but is associated with other specific environmental impacts such as marine resource depletion (32), which was not included here. In general, the effects and interactions of general shifts on bioavailability is a limitation of this study.

Moreover, the life cycle inventories used affect the results obtained. Nutrient quality, if understood as the losses of nutrient quality along the life cycle of a food product or due to storage and preparation methods, is addressed as follows: as closely as possible, the data picked from food tables for each food item represented the food item as it was described in the Dutch diet. For example, if an item was mentioned as “raw vegetable,” then its nutritional composition was taken as such. On the other hand, if a food item was described as “cooked vegetable,” then the food item was chosen as cooked. The purpose of this assessment was not to capture the losses of nutrient quality. Assessing the nutritional composition of food items will always be dependent on data availability in food tables, unless the study actually measures this.

Similarly, as environmental impacts of food products are dependent on country of origin and production system, they are highly variable, which could be a barrier for the extension of this method to other countries. Variability in the results also depends on the methodology used to develop the life cycle inventories. Valuable work is taking place globally among concerned stakeholders (academia, researchers in public and private organizations, agricultural producers, food manufacturers and regulators, to name a few) to harmonize as much as possible those methodologies and to incorporate pragmatically significant developments in environmental science, environmental impact modeling, and big data acquisition and management. All these factors contribute to rapid changes in how the environmental impacts of food products are assessed, understood, and communicated.

Finally, the choices made by the optimization algorithm are only an approximation of actual behavior and the attainability of replacement options.

## Conclusions

We present an innovative method to compare the performance of food groups in a diet: fixing the amounts of a selected food group at different levels and optimizing the remaining diet so that nutritional requirements are met and the changes to the initial diet are minimized. For a set of optimizations we compute environmental (CF, EU, LO) and nutritional metrics (NB). The method is flexible in the number of included nutrient requirements and environmental impacts.

In this paper we compared bread and breakfast cereals, meat, and dairy. Performance of other food groups could be done as an extension of the present study.

The analysis suggested that bread and breakfast cereals are sources of nutrients with a better environmental performance compared to dairy or meat within the context of the Dutch diet. More specifically, amounts of bread and breakfast cereals have an inverse correlation with the environmental impact of the optimized diet. This indicates that they are a source of nutrients with a better environmental performance relative to other foods in the diet. When overall bread and breakfast cereal intake is low in the optimized diet, wholegrain cereal products are the preferred choice due to their nutritional performance.

## Author contributions

GK was the lead researcher in the study, computing, and analyzing the results. EM provided scientific and technical support in the calculations and optimization. NE-O and KC led the application of the Nutrient Balance Concept and its interpretation. MT developed the original optimization algorithm and led the writing and organizing of the final manuscript for scientific publication. HB conceptualized the study and led the Environmental Impact analyzes. All authors were involved in the different drafts and revisions of the article.

### Conflict of interest statement

NE-O and KC were employed by Nestlé Research Center. The remaining authors declare that the research was conducted in the absence of any commercial or financial relationships that could be construed as a potential conflict of interest. This study was partially funded by the Healthgrain Forum, an association, based in Europe, of universities, institutes and industries interested in grain and grain based products. The HGF was not involved in the writing of the article and supported the scientific approach regardless of the results.
