# Supplementary material for: Comparing the Performance of Bread and Breakfast Cereals, Dairy, and Meat in Nutritionally Balanced and Sustainable Diets
Source: Front Nutr. 2018 Jun 7;5:51. doi: 10.3389/fnut.2018.00051 (PMC6002969; doi:10.3389/fnut.2018.00051)
Supplement: Supplementary file 1 [file Table_1.DOCX]

Supplementary Material

Comparing the performance of bread & breakfast cereals, dairy and meat in nutritionally balanced and sustainable diets

Gerard F.H. Kramer, Elsa Valencia Martinez, Namy D. Espinoza-Orias, Karen A. Cooper, Marcelo Tyszler^*^, Hans Blonk

*** Correspondence:** Marcelo Tyszler: mtyszler@gmail.com

# Supplementary Tables

Supplementary Table S1 Lower and Upper daily limits for the optimization; values for the starting diet (not optimized) and 0% bread & breakfast cereals diet (optimized).

| **Property** | **Lower** | **Upper** | **Current diet** | **0%^a^** |
| --- | --- | --- | --- | --- |
| Energy (kcal) | 1994 | 1995 | 1994 | 1995 |
| Protein total (g) | 50 | 125 | 81 | 97 |
| Fat total (g) | 44.4 | 88.9 | 79.0 | 77.2 |
| SAFA (g) | 0.0 | 22.2 | 28.1 | 22.2 |
| PUFA (g) | 0.0 | 26.7 | 15.5 | 18.8 |
| Linoleic acid (g) | 4.4 | - | 13 | 15 |
| ALA (g) | 2.2 | - | 1.7 | 2.2 |
| Trans fatty acids (g) | 0.0 | 2.22 | 1.0 | 0.7 |
| Cholesterol (mg) | 0.0 | 300 | 188 | 218 |
| Carbohydrates total (g) | 200.0 | 350 | 219 | 200.4 |
| Fiber (g) | 30 | - | 19 | 30 |
| Water (g) | 2300 | 3800 | 2979 | 3317 |
| Alcohol (g) | 0.01 | 10 | 6 | 5 |
| DHA+EPA (mg) | 450 | 1000 | 175 | 1000 |
| Retinol act. eq. (μg) | 700 | 3000 | 736 | 803 |
| Vitamin B1 (mg) | 1.1 | - | 0.8 | 1.1 |
| Vitamin B2 (mg) | 1.1 | - | 1 | 1.6 |
| Niacin (mg) | 13 | - | 17 | 23 |
| Vitamin B6 (mg) | 1.5 | 25 | 1 | 1.8 |
| Folate eq. (μg) | 300 | 1000 | 227 | 373 |
| Vitamin B12 (μg) | 2.8 | - | 4 | 10 |
| Vitamin C (mg) | 75 | - | 90 | 165 |
| Vitamin D (μg) | 3.3 | 100 | 2.8 | 3.3 |
| Vitamin E (mg) | 8 | 300 | 13 | 15 |
| Vitamin K total (ug) | 90 | - | 116 | 344 |
| Calcium (mg) | 1000 | 2500 | 1063 | 1097 |
| Phosphorus (mg) | 600 | 3000 | 1500 | 1780 |
| Iron (mg) | 15.0 | 25 | 9.9 | 15.2 |
| Sodium (mg) | 0.0 | 2400 | 2363 | 2340 |
| Potassium (mg) | 3100 | - | 3223 | 4687 |
| Magnesium (mg) | 280 | 530 | 325 | 467 |
| Zinc (mg) | 7 | 25 | 10 | 12 |
| Selenium (μg) | 50 | 300 | 44 | 80 |
| Iodine (μg) | 150 | 600 | 160 | 150 |

^a^ Values in orange are out of bounds. Those in yellow are at the boundary.
